# The Pharmaceutical Industry in 2016. An Analysis of FDA Drug Approvals from a Perspective of the Molecule Type

**DOI:** 10.3390/molecules22030368

**Published:** 2017-02-27

**Authors:** Beatriz G. de la Torre, Fernando Albericio

**Affiliations:** 1 School of Laboratory Medicine and Medical Sciences, University of KwaZulu-Natal, 4001 Durban, South Africa; 2 School of Chemistry, University of KwaZulu-Natal, 4001 Durban, South Africa; 3 CIBER-BBN, Networking Centre on Bioengineering, Biomaterials and Nanomedicine, University of Barcelona, 08028 Barcelona, Spain; 4 Department of Organic Chemistry, University of Barcelona, 08028 Barcelona, Spain

**Keywords:** drug discovery, API, peptide, biologics, small molecules, chemical entities

## Abstract

This is an analysis from a chemical point of view of the 22 drugs accepted by the FDA during 2016. The different drugs from the 2016 “harvest” have been classified according to their chemical structure: antibodies; TIDES (oligonucleotides and peptides); amino acids and natural products; drug combination; and small molecules.

Health is possibly one of the most “magical” words associated with the development of the human being. From the most ancient societies to the present, restoring or improving social health and wellbeing has been a major pursuit. Although the use of traditional medicines, based mainly on the use of natural products treated with simple manipulations, continues to be common in some communities worldwide for the treatment of some diseases, the last century witnessed the birth of the pharmaceutical industry [1].

The pharmaceutical sector is, without a doubt, the most intriguing industrial activity. At a glance, it is associated with heavy investments, strong regulations, and a low number of outputs in terms of new products [2]. During the last 20 years (1997–2016), the U.S. Food and Drug Administration (FDA), which is probably the most important regulatory agency, followed by the European Medicines Agency (EMA), has approved a total of 595 new entities [3,4]. Of these, 491 were new chemical entities (NCEs) and 103 biologics, the latter accounting for 17% of the total (Figure 1). The pharmaceutical industry produces approximately 30 new entities yearly—25 being NCEs and five biologicals.

With this relatively low number of new products in the market, an analysis of the new entities approved by the FDA performed at the end of the year can reflect the “health” of the sector.

In 2016, a total of 22 new entities were approved by the FDA—of these 15 NCEs and seven biologicals. These figures caused some surprise among analysts [4,5], because in 2015 and 2014 the same agency approved 45 (33 + 12) and 41 (30 + 11), respectively. These last figures were the first and third best in the two decades. Analysts interpret these numbers with caution, because it is important to take into account that launching a new drug into the market is slow process that can take an average of between 10 and 15 years. Therefore delays in drugs reaching the market can result from the intrinsic dynamics of the process itself and from associated players such as GMP manufacturers, because it should be born in mind that the production of many of the new drugs is highly challenging.

On the basis of the analysis of FDA figures in 2016, it can be concluded that the niche occupied by biologics (seven monoclonal antibodies) is growing. In this regard, 2016 (Table 1) was the third best year after 2015 (12) and 2014 (11), with biologics accounting for 32% of the total for the year, which is the highest figure in two decades. Investments in this field are increasing. In this regard, the development of antibody drug conjugates (ADCs) and proteins ensures an increase in biologics production. 

An analysis of the FDA data on NCEs (which until just a few years ago were primarily small molecules) for 2016 reveals that three oligonucleotides were approved (Figure 2). Thus, the approval of Spinraza, a 18-mer antisense oligonucleotide targeting spinal muscular atrophy (Figure 2B); Exondis 51, a 30-mer morpholino phosphorodiamidate antisense oligomer against Duchenne muscular dystrophy (Figure 2C); and Defitelio, a mixture of oligodeoxyribonucleic acids for hepatic veno-occlusive disease (Figure 2D), marks the advancement of these chemical species and implies a returns on the large investments made by the pharmaceutical industry in this field since the earlier 1990s.

Other TIDES (oligo- pep-TIDES) approved this year include Adlyxin, a 44-amino acid peptide antidiabetic (Figure 3), with a molecular weight of 4858 that belongs to the family of glucagon-like peptide-1 (GLP-1) agonist. Specifically, Adlyxin is derived from exendin-4, where the Pro at the C-terminal has been removed and a linker of Lys_6_ (Figure 3, highlighted Blue) has been added instead [6].

These four biomolecules are a clear example of the power of solid-phase synthesis for the industrial production of TIDES-based Active Pharmaceutical Ingredients (APIs). Only a few years ago, the pharmaceutical industry was reluctant to consider peptides and oligonucleotides of this size as potential drugs, because of the lack of reliable chemical strategies for their preparation. However, the synthetic advances using the solid-phase methodology now makes the production of these large TIDES-based APIs feasible. 

From a chemical point of view, it is interesting to highlight Xiidra (for dry eye disease), because it is a acyl dipeptide formed by two unnatural amino acids, 3-methylsulfonylphenylalanine (Figure 4A, blue) and 5,7-dichloro-1,2,3,4-tetrahydroisoquinoline-6-carboxylic acid (Figure 4A, orange), whose amine is acylated with benzenebenzofuran-6-carboxylic acid.

Briviact (epilepsy) is a compound derived from the amino acid α-ethylglycinamide (Figure 4B, blue). Continuing with amino acids, Alumni is an α,α-disubstitutedglycine (Figure 4C, blue) bearing an ^18^F atom and used as a diagnostic agent in positron emission tomography (PET) imaging for prostate cancer. In this same class, Netspot has been approved as a kit for the preparation of ^68^Ga dotatate injection for PET scans. In the field of natural product-based drugs, Ocaliva (primary biliary cholangitis), which is a semisynthetic bile acid, has been also approved (Figure 4D).

Zepatier (Figure 5A) and Epclusa (Figure 5B) are two two-drug combinations for the treatment of hepatitis C. Both contains a hepatitis C virus NSSA inhibitor (Elbasvir and Velpatasvir, respectively) and Grazopevir (macrocycle, NS3/4A protease inhibitor) and Sofosbuvir (nucleotide, viral RNA polymerase inhibitor), respectively.

The last four compounds: Rubraca an indole derivative for the treatment of patients with deleterious BRCA mutation associated to ovarian cancer (Figure 6A); Eucrisa, a hemiboronate for atopic dermatitis (Figure 6B); Nuplazid, which acts in some psychotic disorders in Parkinson’s disease, and is a trisubstituted urea (Figure 6C); and Venclexta, a complex sulfonamide used in the treatment of chronic lymphocytic leukaemia (Figure 6D), are those that best fit the small molecule category, although the last one has a molecular weight of 868. 

In conclusion, while the number of drugs approved by the FDA in 2016 was smaller than expected, taking into account preceding years, they show great diversity from a chemical point of view. In this regard, the most important highlight is probably the presence of the three oligonucleotides, which are expected to open up new avenues for this key category of biomolecule. Advances in the solid-phase methodology for the preparation of large TIDES are driving the development of these kinds of biomolecule as APIs [7]. The approval of seven monoclonal antibodies is also worth noting. There is no doubt that this class of biologics is the most suitable for the treatment of hitherto intractable diseases. More ADCs are expected to be approved in the coming years, thus positioning antibodies as first class pharmaceuticals. The outputs from 2016 indicate the so-called small molecules are losing ground against biologics, biomolecules, and other molecules inspired on natural products. However, the ultimate aim of any drug, regardless of the chemical species, is to bring relief to those suffering. If this is achieved, many will benefit.

## Figures and Tables

**Figure 1:**
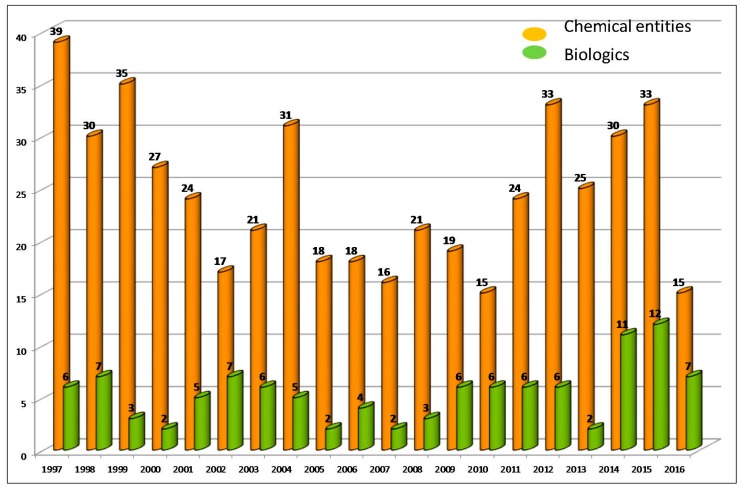
New Chemical Entities and Biologics approved by the FDA in the last two decades [3,4].

**Figure 2:**
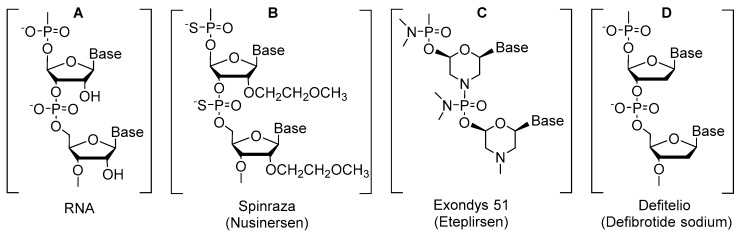
General structure of oligonucleotide-based drugs. Standard RNA strand (**A**); Phosphorothioate backbone (**B**); Morpholino Phosphorodiamidate backbone (**C**); Standard DNA strand (**D**).

**Figure 3:**
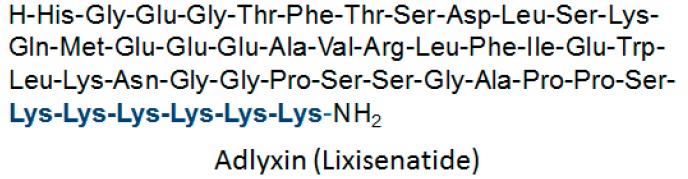
Structure of Adlyxin.

**Figure 4:**
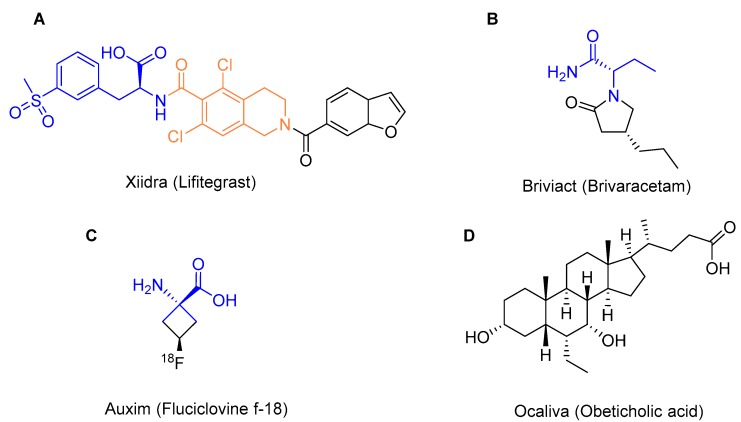
Structures of Xiidra (**A**); Briviact (**B**); Auxim (**C**); and Ocaliva (**D**).

**Figure 5:**
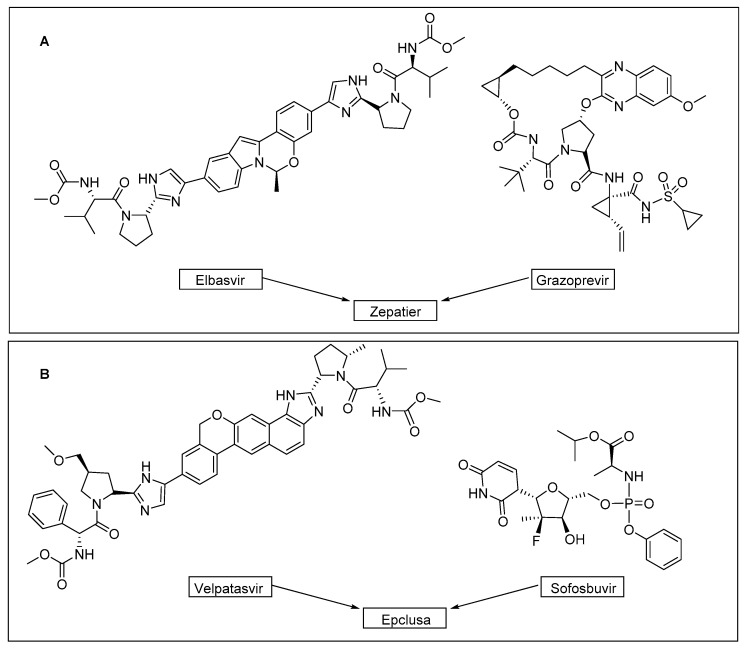
Structures and composition of Zepatier (**A**) and Epclusa (**B**).

**Figure 6:**
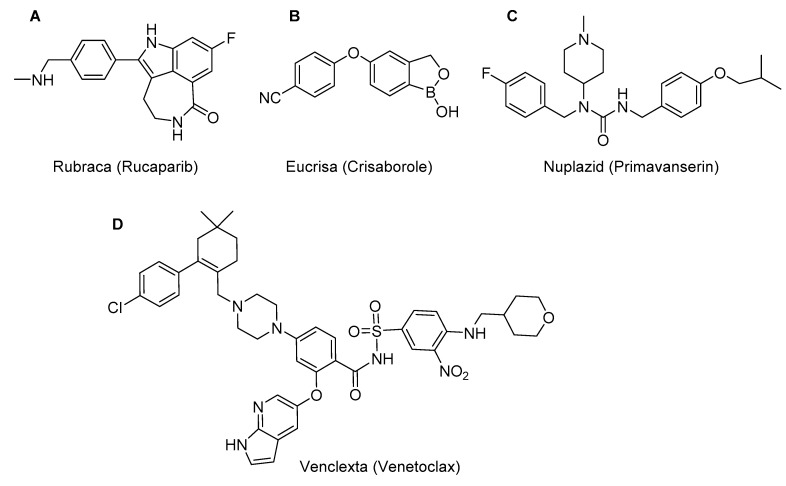
Structures of Rubraca (**A**); Eucrisa (**B**); Nuplazid (**C**); and Venclexta (**D**).

**Table 1:** Monoclonal Antibodies approved by the FDA during 2016 ^a^.

Trade Name	Active Ingredient	Target/Disease
Anthim	Obiltoxaximab	Anthrax
Cinqair	Reslizumab	Severe asthma
Lartruvo	Olaratumab	Soft tissue sarcoma
Taltz	Ixekizumab	Plaque psoriasis
Tecentriq	Atezolizumab	Urothelial carcinoma
Zinbryta	Daclizumab	Multiple sclerosis
Zinplava	Bezlotoxumab	*C. difficile* recurrence

^a^ Source FDA.
